# A novel quantification platform for point-of-care testing of circulating MicroRNAs based on allosteric spherical nanoprobe

**DOI:** 10.1186/s12951-020-00717-z

**Published:** 2020-10-31

**Authors:** Huiyan Tian, Changjing Yuan, Yu Liu, Zhi Li, Ke Xia, Mengya Li, Fengxin Xie, Qinghai Chen, Ming Chen, Weiling Fu, Yang Zhang

**Affiliations:** 1 Department of Laboratory Medicine, First Affiliated Hospital, Third Military Medical University (Army Medical University), Chongqing, China; 2 Department of Laboratory Medicine, Chongqing University Cancer Hospital, Chongqing, China

**Keywords:** Allosteric spherical nanoprobe, Dual-hairpin, MiRNA-150, Förster resonance energy transfer (FRET), Point-of-care testing (POCT)

## Abstract

MiRNA-150, a gene regulator that has been revealed to be abnormal expression in non-small cell lung cancer (NSCLC), can be regarded as a serum indicator for diagnosis and monitoring of NSCLC. Herein, a new sort of nanoprobe, termed allosteric spherical nanoprobe, was first developed to sense miRNA-150. Compared with conventional hairpin, this new nanoprobe possesses more enrichment capacity and reaction cross section. Structurally, it consists of magnetic nanoparticles and dual-hairpin. In the absence of miRNA-150, the spherical nanoprobes form hairpin structure through DNA self-assembly, which could promote the Förster resonance energy transfer (FRET) of fluorophore (FAM) and quencher (BHQ1) nearby. However, in the presence of target, the target-probe hybridization can open the hairpin and form the active “Y” structure which separated fluorophore and quencher to yield “signal on” fluorescence. In the manner of multipoint fluorescence detection, the target-bound allosteric spherical nanoprobe could provide high detection sensitivity with a linear range of 100 fM to 10 nM and a detection limit of 38 fM. More importantly, the proposed method can distinguish the expression of serum miRNA-150 among NSCLC patients and healthy people. Finally, we hoped that the potential bioanalytical application of this nanoprobe strategy will pave the way for point-of-care testing (POCT). 
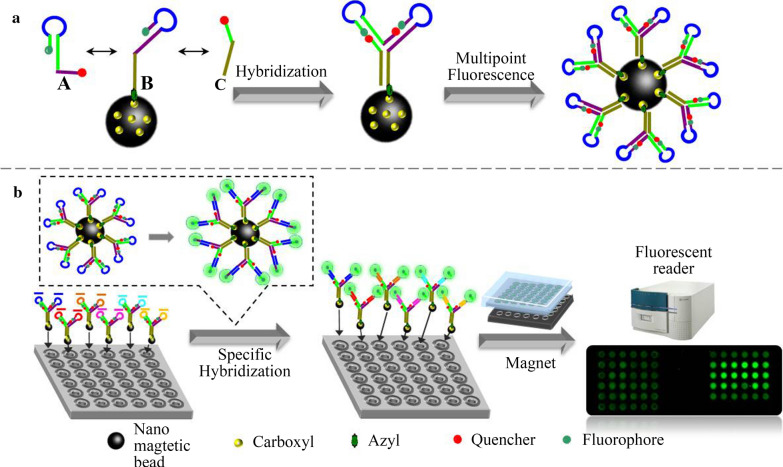

## Introduction

MicroRNAs (miRNAs) are sorts of small single stranded noncoding RNAs and its length are approximately 21 ~ 25 nucleotides [1–3]. Since the dysregulation of miRNAs is closely related to the occurrence and prognosis of tumor, the detection of circulating miRNAs in serum can be used as credible biomarkers for tumor liquid biopsy [4, 5]. For instance, monitoring the circulating miRNA-150 in serum could provide reliable clinical assays for diagnosis early non-small cell lung cancer (NSCLC) [6]. Thus, the sensitive and specific detection of miRNAs is considered to be a meaningful strategy for early cancer diagnosis.

Currently, the detection of miRNAs is normally performed by traditional techniques such as northern blotting, microarray and reverse transcription-polymerase chain reaction (RT-PCR) [7–9]. Although these methods are reliable, they are hardly applied to clinic due to the disadvantages of complicated operations, high cost, unstable results and required well-trained staff. Certainly, other various techniques have been developed, including electrochemical sensor, surface plasmon resonance (SPR), surface-enhanced Raman scattering (SERS), colorimetry and fluorescence [10–14]. Among the various techniques, fluorescence detection platform has emerged as an excellent alternative for the detection, quantification and characterization of target [15–17]. Organic fluorescent dye-labeled DNA probe and fluorescent technique have been widely employed for ultrasensitive quantification of DNA, miRNA, proteins, and others because of the easily commercialized synthesis [18–20]. To upgrade the assay sensitivity, most of these determinations were carried out assist with signal amplification strategies, including rolling circle amplification (RCA), exponential amplification reaction (EXPAR), catalytic hairpin assembly (CHA), entropy driven catalytic reaction, enzyme-assisted signal amplification and materials, etc. [21–26]. However, these amplified methods also suffered from various drawbacks and limitations in practice, such as long time and high-budget needed, reaction condition rigorous, not well met the demand of universal simple and rapid medical analysis, especially for point-of-care testing (POCT). Therefore, it is expected to establish one kind of simpler method for target directly detection without the help of any amplification strategy.

One possible solution is that the allosteric spherical nanoprobes have high reaction cross section and show high binding capacity and specificity to target miRNA. Up to now, the hairpin probe has always been attracted particular interest as the simplest, prototypical system. Tyagi and Kramer were the pioneers to study hairpin shaped molecular probe [27]. The nature of molecular beacon is single-stranded DNA molecule, which contains a stem-and-loop structure and a pair of fluorophore and quencher group. The loop is design to hybridize with its complete complementary target. And the stem is the result of two complementary arm sequences through annealing. Due to the proximity of a pair of fluorophore and quencher group, the fluorescence was quenched. So, when the loop recognizes the perfectly target, the structure of hairpin changes into a more stable formation of DNA duplex and forces the stem apart, resulting in the leakage of fluorescence. To date, various molecular probe-based detection methods have been proposed in a large scale of applications. For example, the detection of DNA damage, the monitoring of DNA movement, biological small molecule detection, and miRNA detection in living cells [28–33]. Nevertheless, molecular probe-based nucleic acids detection is also generally on the basis of the above-mentioned amplification techniques.

Here, we design a unique allosteric spherical nanoprobe based on the traditional molecular beacon idea. Fluorescent groups and quenching groups are respectively marked at the end-to-end joint of the two hairpin structures. Numerous dual-hairpin structures are modified on the surface of magnetic nanoparticles. So, the nanoprobe was endowed with enrichment capacity and reaction cross section so as to be easier to react with the target. When the target exists, the closed dual-hairpin spherical nanoprobe is opened to active “Y” structure, leading to significant fluorescence leakage. Following that, the results of fluorescence were recorded on multipoint fluorescence scanning microarray. The manner of fluorescence readout is different from traditional fluorescent scanner, the microarray device is more portable and compact, especially for POCT application. In this way, the miRNA-150 detection is determined by the fluorescence changes in the allosteric spherical nanoprobe upon binding with the target. Due to the inherent fluorescence signal transduction mechanism, the dual-hairpin spherical structure functions as a sensitive probe with a high signal-to-background ratio. Meanwhile, this allosteric spherical nanoprobe has high hybridization specificity because of its loop-stem structure, which can easily discriminate the complementary from single-mutation target miRNA.

## Material and methods

### Reagents and materials

Polyacrylamide gel electrophoresis (PAGE)-purified DNA oligonucleotides and miRNAs were manufactured by Takara Biotechnology Co., Ltd. (Dalian, China, https://www.takara.com.cn/). These sequences are summarized in Additional file 1: Table S1. Carboxylic modified nanometer magnetic beads (100 nm) were obtained from huier Nano Science and Technology Co., Ltd. (Henan, China, https://www.huierbio.com/). MiRNA Extraction Kit (Blood), TaqMan miRNA cDNA Synthesis Kit, TaqMan hsa-miR150 qPCR Kit and TaqMan hsa-miR16 qPCR Kit were supplied by HaiGene Biotechnology Co., Ltd. (Harbin, China, https://www.haigene.cn/contact.html). PAGE Gel Silver Staining Kit and staining dye of 10,000 × Sybr green I was purchased from Solarbio Science & Technology Co., Ltd. (Beijing, China, https://www.solarbio.com/). 1-(3-Dimethylaminopropyl)-3-ethylcarbodiimide hydrochloride (EDC), and 2-(N-Morpholino) ethanesulfonic acid (MES) were provided by Sigma Aldrich (St. Louis, MO, USA, https://www.sigmaaldrich.com/china-mainland.html). 5 × TBE (450 mM Tris-boric acid, 10 mM EDTA, pH 8.0) and 10 × TNE buffers were bought by Leagene Biotechnology (Beijing, China, https://leagene.bioon.com.cn/). 20 bp DNA ladder and miRNA marker were ordered from Takara Biotechnology Co., Ltd. (Dalian, China, https://www.takara.com.cn/) and New England Biolabs Co., Ltd. (Beijing, China, https://www.neb-china.com/) respectively. To avoid the hydrolysis of target sequences by RNase, 0.1% DEPC treated sterilized water was used through the entire experiment. The RNase-free tubes and tips were supplied by Axygen Scientific Inc. (Silicon Valley, USA, https://axygen.bioon.com.cn/). All chemicals employed were of analytical reagent grade. High-purity deionized water prepared by a Milli-Q water purification system (Millipore Corp., Bedford, USA) was used for all experiments.

### Apparatus and instruments

NanoDrop 2000c spectrophotometer (Thermo, USA, https://www.thermofisher.com/cn/zh/home.html) was used to qualified and purity of total miRNAs. LuxScan 10 K Microarray Scanner (CapitalBio Technology, https://www.capitalbiotech.com/) and Varioskan LUX Multimode Microplate Reader (Thermo, USA, https://www.thermofisher.com/cn/zh/home. html) were used for fluorescence detection and signal output. Gel image was carried on Gel Doc XR + Imaging System (Bio-Rad, USA, https://bio-rad.biomart.cn/). Fluorescence quantitative PCR (Bio-Rad, USA, https://bio-rad.biomart.cn/) was used for quantitative analysis of nucleic acid samples and monitoring the fluorescence intensity changes in hybrid reaction systems. TMM-5 M Magnetic programmable mixer (Topscien, China, https://www.topscien.com/) was used to mix liquid samples. All the glassware needed in the experiment were flushed and silicified with doubly-steamed water.

### Design of microarray structure

Glass chips with 6 × 6 microarray were customized by Zhenjiang Huarui chip technology Co., Ltd. The size of the chip was 75 mm × 25 mm × 1.1 mm, the diameter of the sample pool was 2 mm, and the depth was 0.8 mm (Additional file 1: Fig S1). All the size of microarray chip was in accordance with the detection module of the LuxScan 10 K Microarray Scanner.

### Synthesis of dual-hairpin spherical probe

First, electrolytic ions and aggregating particles impurities in nanometer magnetic beads were removed by nitric acid fiber film filter with 0.22 μm. After ultrasonic treatment, 1 mL of carboxyl magnetic nanoparticles were blended and washed twice with 1 mL of MES (15 mM, pH 6.0, 4 °C) to avoid the agglomeration. Afterwards, the magnetic nanoparticles were sinned and adsorbed with magnetic separator and the supernatant was removed. Next, 100 μL of MES and 100 μL of EDC (10 mg/mL) were added to the magnetic nanoparticles. The solution was blended completely at room temperature for 30 min to suspend the magnetic nanoparticles and activate the carboxyl. Later, the supernatant was removed again. The activated carboxyl magnetic nanoparticles could be used for coupling. Meanwhile, the prepared fixed chain (Seq B) was diluted to wanted concentration with MES. Afterwards, the fixed aggregation chains (200 μL of the diluted Seq B) of the dual-hairpin probes modified with azyl were covalently coupled with the carboxyl magnetic nanoparticles. This process was completed by TMM-5 M Magnetic programmable mixer overnight at room temperature. So far, the carboxyl magnetic nanoparticles coupled with Seq-B were ready for following experiments and bioassays.

### Fluorescence detection

Fluorescence detection was performed at LuxScan 10 K Microarray Scanner and Varioskan LUX Multimode Microplate Reader. Fluorescence emission spectra was collected in the range of 500 to 650 nm with an excitation wavelength of 490 nm and slit width of 5.0 nm.

### Gel electrophoresis

The non-denaturing 12% polyacrylamide gel electrophoresis (PAGE) analysis was carried out for 120 min in 1 × TBE buffer at pH 8.3 under 90 V constant voltage. 15 μL of sample and 3 μL of loading buffer were completely mixed, and the concentrations of each sample were both 2000 nM. Two ladders were used to indicate the molecule weight, one was 20 bp DNA ladder and the other was miRNA marker. Then, the gel was stained with Sybr green I for 30 min and then imaged using a Bio-Rad Gel Doc XR + Imaging System (Bio-Rad, USA).

### Preparation of clinical plasma samples

All samples were obtained from patients at the Southwest Hospital of Third Military Medical University (Army Medical University) with ethical approval. There were 7 patients (male 4, female 3) with average age of 50.23 ± 4.25 years in NSCLC group. In control group, 3 patients (male) with average age of 49.16 ± 4.13 years were involved. Any patient with insufficiency of liver or renal function, malignancy, peripheral vascular disease, and blood individual was not found in the two groups.

Each sample of blood (300 μL) was cracked with lysis solution, then extracted by ethanol and isopropanol. After absorbing the total miRNA by spin column, 30 μL of the RNase free TE buffer was added to elute the miRNA. The above-mentioned miRNA products were reverse transcribed and the cDNAs were stored at − 80 °C.

### Extraction of circulating miRNAs and expression profile analysis of miRNA-150

According to the manufacturer’s procedure, circulating miRNAs were extracted from anticoagulation of peripheral blood. The RT-PCR was performed on a Thermal Cycler 2400 and the Q-PCR was on a CFX-96. Real-time PCR profiling was performed with the following cycling conditions: 95 °C for 15 min; 40 cycles of 95 °C for 10 s, 60 °C for 60 s. Besides, the total 20 μL for Q-PCR reaction mixture contained 2.5 μL of cDNA, 4 μL of 5 × Golden HS TaqMan qPCR Mix, 1 μL of 20 × miRNA TaqMan Assay and 12.5 μL of RNase-free water.

### Analysis of data

In the profiling experiment, miRNA expression data were normalized toward the average expression of miRNAs detectable in all samples. By strictly controlling of the amplification conditions and primers, the efficiency of the PCR was close to 1. Fold changes of relative expression were calculated using the △△C_T_ method, the − △△C_T_ values were calculated by using the formula “2^− △△CT^ = [(C_T_ gene of interest–C_T_ internal control) sample A–(C_T_ gene of interest–C_T_ internal control) sample B]”. Thus, higher scores represent higher expression levels [34]. All data were analyzed with SPSS 18.0. Significance and very significance were accepted as P < 0.05 and P <  0.01, respectively.

## Results and discussion

### Design principle of allosteric spherical nanoprobe

**Scheme 1:**
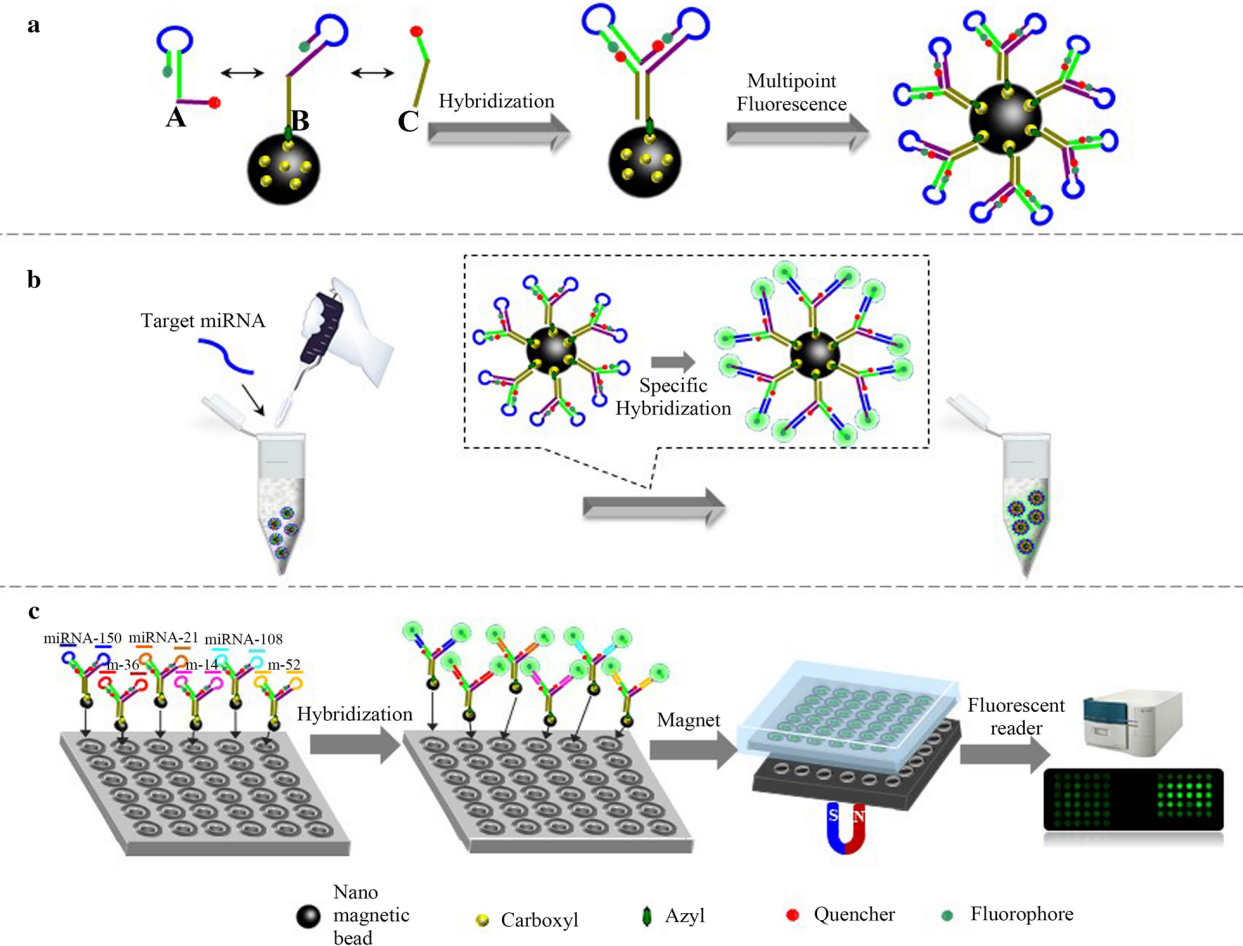
Schematic illustration of the allosteric spherical nanoprobe for miRNA-150 direct multipoint fluorescence detection

As shown in Scheme 1, the allosteric spherical nanoprobe was structurally consisted of three sequences (Seq A: the free chain, Seq B: the fixed chain, Seq C: the auxiliary chain) and formed double rings that were specifically hybridized with target nucleic acids. In this magnetic nanoparticles allosteric spherical probe system, the FAM and BHQ1 are respectively marked at the end-to-end joint of the two hairpin structures. Then, numerous dual-hairpin structures are conjugated to the surface of magnetic nanoparticles. When no miRNA-150 exists, the spherical nanoprobe forms firmly hairpin structure by intramolecular hybridization, which could promote the FRET of FAM and BHQ1 nearby. However, in the presence of target, the target-probe hybridization can specifically open the hairpin and form the active “Y” structure which separated fluorophore and quencher to yield an “active” fluorescence. Subsequently, this complex can be precipitated to the bottom of the microarray chip with the aid of magnet. Finally, the results of fluorescence were recorded on multipoint fluorescence scanning microarray. In this manner, the target-binding allosteric spherical nanoprobe leads to significant fluorescence signal enhancement and the fluorescent intensity is related to the concentration of target miRNA-150. The design of double rings makes it easier to detect target than traditionally one hairpin DNA probe due to the magnet nanoparticles enrichment numerous dual-hairpin probes and increase the larger reaction cross section accordingly. Besides, the reaction cross section of the ring sequence is larger than that of the stem sequence, which ensures that the target sequence can be combined with the ring sequence to form a more stable structure of DNA double strand and then pull the stem apart. Meanwhile, each of the three sequences mismatched completely, thus avoiding the formation of non-specific hybridization and ensuring the sufficiently FRET between fluorophore and quencher. In principle, the concept of integrating numerous dual-hairpin probes on a single magnetic nanoparticle has made it easier to expand the functionality of this nanoprobe.

### Verify the structure of the allosteric spherical nanoprobe

**Fig. 1:**
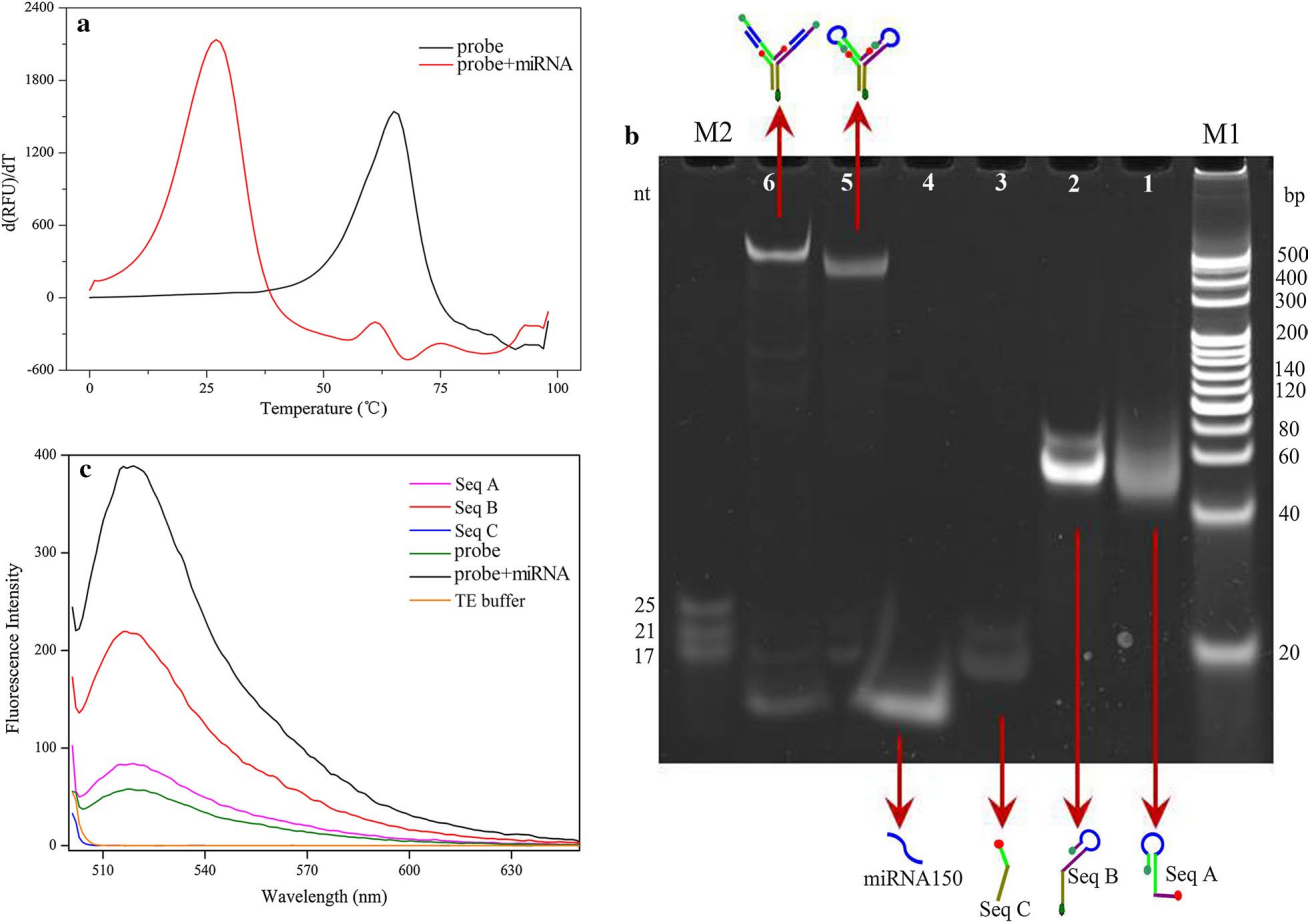
Characterization of the allosteric spherical nanoprobe. **a** Melting curve of allosteric spherical nanoprobe and the hybridization structure. **b** Non-denatured PAGE analysis of allosteric spherical nanoprobe. Lane 1, Seq A; lane 2, Seq B; lane 3, Seq C; lane 4, miRNA-150; lane 5, dual-hairpin; lane 6: dual-hairpin and miRNA-150; M1, DNA marker; M2, miRNA marker. **c** Fluorescence emission spectra of different sequence in allosteric spherical nanoprobe. The concentrations of samples were both 2000 nM

A stable allosteric spherical nanoprobe structure is one of the prerequisite for the success of this novel approach. For the sake of verifying the spherical nanorobe structure, the following experiments were conducted. Firstly, considering that the high-resolution melting can reflect the dissociation temperature, after degeneration at 98 °C, the melting curve from 98 °C to 0 °C was analyzed in Fig. 1a. Obviously, each group has different and unique melt peak from each other, indicating that a single hybrid structure was formed and no dimer was produced. Secondly, the non-denaturing PAGE (12%) analysis was performed to verify the structure of the allosteric spherical nanoprobe. In Fig. 1b, lane 5 showed that the intramolecular hybridization allosteric spherical nanoprobe consists of Seq A, Seq B and Seq C had slower mobility owing to the higher molecular mass than the three single sequence from line 1 to lane 3 and lane 4 (miRNA-150). Besides, the reaction products of allosteric spherical nanoprobe and miRNA-150 were obtained in lane 6 with lower migration rate than lane 5. These results confirm that the allosteric spherical nanoprobe structure was initially successfully constructed and can react with the target. Thirdly, the fluorescence emission spectra in Fig. 1c clearly indicated that the low fluorescent intensity of this allosteric spherical nanoprobe and other four single sequence. This phenomenon is the expected hairpin intramolecular hybridization result. When the target was introduced, higher fluorescence intensity was appeared, indicating the target could bind with allosteric spherical nanoprobe and change the structure of the hairpin thereby generating significant fluorescent signal.

### Feasibility of the allosteric spherical nanoprobe

**Fig. 2:**
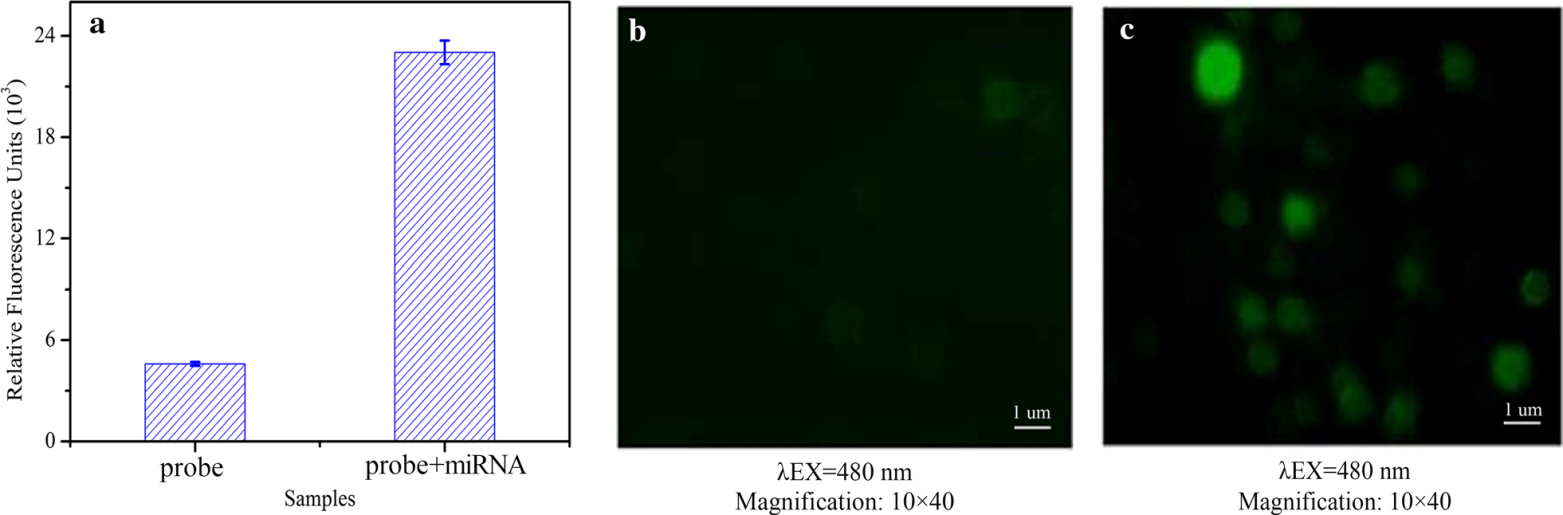
Feasibility of allosteric spherical nanoprobe assay. **a** Fluorescence intensity of allosteric spherical nanoprobes in the absence and presence of miRNA-150. **b** Fluorescence micrograph image of hairpins-modified magnetic beads. **c** Fluorescence micrograph image of reaction products of allosteric spherical nanoprobes and miRNA-150

The allosteric spherical nanoprobe was design to combine with miRNA-150 specifically and turn the “inactive” hairpin probe to “active” fluorescent state. So, the fluorescence measurement was firstly carried out to verify the feasibility of the developed method. As depicted in Fig. 2a, the fluorescence intensity was very weak in control group without the target, low background signal may be attributed to the perfect combination between FAM and BHQ1 at the end-to-end joint of the two hairpin structures. In contrast, there was a strong fluorescence intensity appeared when miRNA-150 existed in the allosteric spherical nanoprobe system. Furthermore, the results of fluorescence microscope before and after the reaction between the allosteric spherical nanoprobe and the target are consistent match with those of fluorescence. As illustrated in fluorescence microscope field of view (Fig. 2b), almost no fluorescent dot was shown when no target was existed, contributing the intramolecular hybridization of allosteric spherical nanoprobe. Interestingly, we observed that the fluorescent spots, the reaction products of hairpins and targets, full filled the field of vision of a fluorescent microscope (Fig. 2c). The data suggested that the target reacted with allosteric spherical nanoprobe successfully, resulting in obvious fluorescence leakage. Therefore, it is reasonable to believe that the feasibility of the allosteric spherical nanoprobe is workable.

### Optimum condition of hybridization experiment

**Fig. 3:**
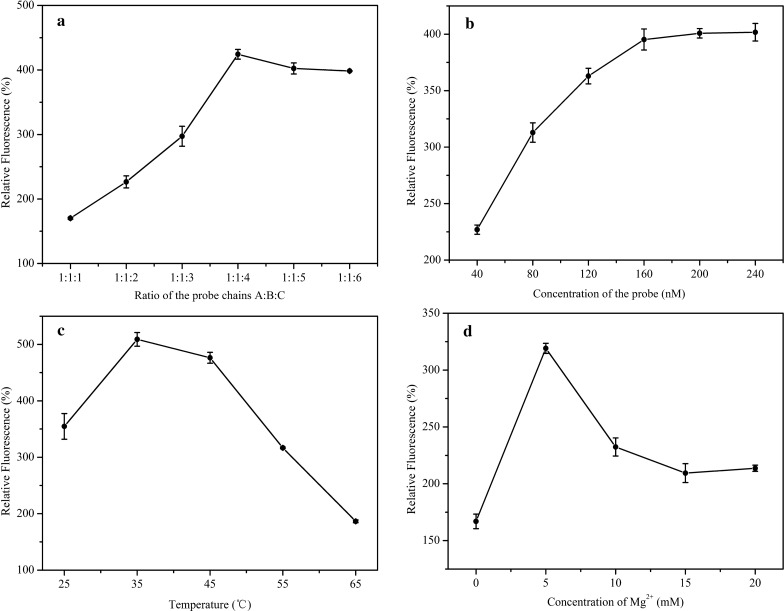
Optimization of allosteric spherical nanoprobe assay. **a** The ratio of different components in probe (A:B:C). **b** The ratio of microsphere to probes. **c** The reaction temperature. **d** The concentration of Mg^2+^

The components in the allosteric spherical nanoprobe such as Seq A, Seq B and Seq C were indispensable and played essential roles in target detection. Subsequently, the ratio of different components (Seq A, Seq B and Seq C) in probe and the concentration of probes, the reaction temperature and the concentration of Mg^2+^ were studied to obtain the best analytical performance for this detection system. Prior to construct this probe, not only should a proper hairpin structure be ensured, but also fluorescent leakage among Seq A and Seq B and Seq C should be avoided. As can be seen from the data in Fig. 3a, the sufficient amount of Seq C could ensure the realization of FRET during hybridization, while the excessive Seq C would not affect the hairpin probe and the free Seq C in solution would be removed by magnetic separation, the relative fluorescence intensity increased with the increasing ratio of the components and become plateau from 1:1:4. Thus, 1:1:4 was chosen as the optimal concentration ratio among Seq A, Seq B and Seq C.

Similarly, the carboxylation sites on the magnetic nanoparticles and the corresponding amount of hairpins bound to them are considered to be factors affecting the probe performance. So, the concentration of probes was discussed. As shown in Fig. 3b, with the increase concentration of probe, the relative fluorescence intensity gradually increased until reaching a relatively stable at 160 nM, indicating that the optimal concentration of the probe is 160 nM. This optimization result revealed that excess probes couldn’t participate in the reaction due to two respects reasons. One is the steric effects of hairpin structure made the probes cannot completely bind to the carboxylation site on the surface of magnetic nanoparticles. The other is the carboxylation sites on the surface of magnetic nanoparticles were completely bound with probes. So, redundant probes are not affect the magnetic bead based probes system. Finally, 160 nM was regarded as the proper concentration of the allosteric spherical nanoprobe.

In order to meet the demands of ideal reaction temperature in this probe system, different reaction temperatures were tested to gain the best analysis fluorescent signals. As depicted in Fig. 3c, the relative fluorescence intensity reached its peak at 35 °C, and decreased rapidly as the reaction temperature increased. This trend indicated that, with the increase of reaction temperature, the structural stability of allosteric spherical nanoprobe decreased and the relative fluorescence signal of allosteric spherical nanoprobe decreased accordingly. At last, the optimal temperature was determined at 35 °C for the sake of the stability of the probe structure and the performance of this probe system.

Since different ionic strengths will affect the melting temperature of probe and a certain ionic strength can stabilize the stem hairpin structure [35], Zhang’s group have verified that the stem structure of the molecular beacon was opened when there was no Mg^2+^ in the buffer [36]. So, the concentration of Mg^2+^ associated with the stability of allosteric spherical nanoprobe was further investigated. As exhibited in Fig. 3d, the relative fluorescence intensity reached the maximum when the concentration of Mg^2+^ was 5 mM, indicating that hairpin structure maintains a good stable state. While excessive dosage of Mg^2+^ may influence the identification and hybridization efficiency between dual-hairpin and target. Therefore, 5 mM was selected as the satisfactory concentration of Mg^2+^ in the following experiment.

### Performance of the allosteric spherical nanoprobe

**Fig. 4:**
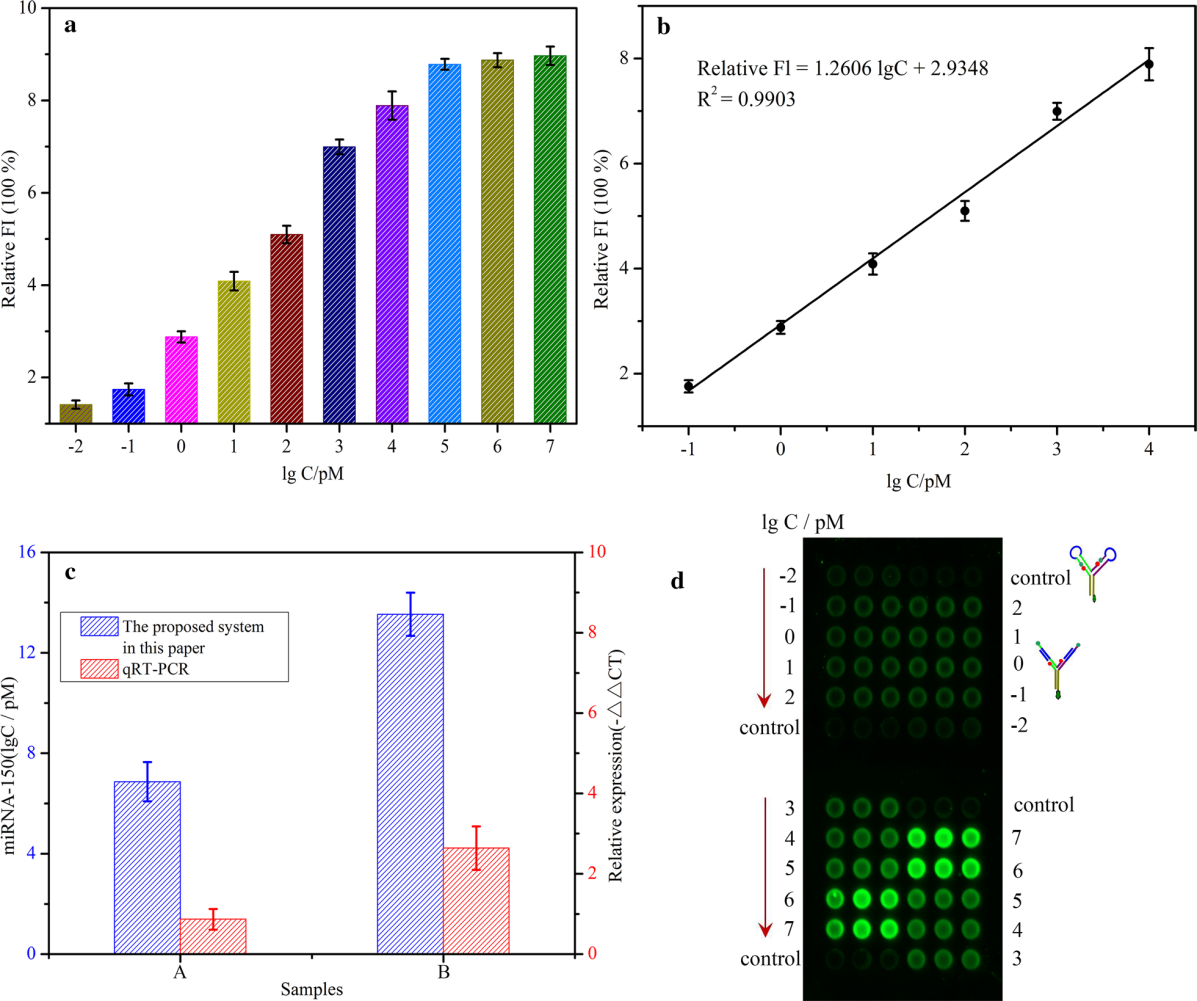
Allosteric spherical nanoprobe assay performance for miRNA-150 detection. **a** The relative fluorescence intensity of the allosteric spherical nanoprobes with different miRNA-150 concentrations. **b** The linear relationship between the relative fluorescence intensity and the logarithm of miRNA-150 concentration over the range from 100 fM to 10 nM. **c** The results of real human serum samples for both NSCLC cancer patients (B) and healthy donors (A) with qRT-PCR (red) and the proposed method (blue). **d** The multipoint fluorescence scanning results on microarray corresponding to (**a**)

Under the selected experimental conditions, the performance of the proposed method for quantitative analysis of miRNA-150 was further investigated by detecting targets with different concentrations. Figure 4a expressed the relative fluorescence intensity of miRNA-150 with different concentrations in this allosteric spherical nanoprobe system. The higher the target concentration, the stronger the fluorescence intensity. Figure 4b revealed a good linear relationship between the relative fluorescence intensity and the logarithm of miRNA-150 concentration over the range from 100 fM to 10 nM. The linear regression equation was *Relative FI* = 1.2606 lg*C* + 2.9348, with a correlation coefficient of *R*^*2*^ = 0.9903. The threshold of detection limit was set to three-fold standard deviation above blank. Therefore, the detection limit was estimated as 38 fM. Correspondingly, the multi-point fluorescence scanning results on microarray could be seen in Fig. 4d. The wide linear range and low detection limit reflect the quiet good performance of this strategy. Additionally, this proposed method was comparable to some previously reported fluorescence sensors (Additional file 1: Table S2) with pretty simple design, good sensitivity and relative short detect time for miRNAs detection [19, 37–41]. This method is characterized by the ingenious design of a magnetic bead-based allosteric spherical nanoprobe, which has stronger enrichment capability and larger reaction cross section for the target.

### Detection of miRNA-150 in serum samples

To identify the performance of the new method for detecting miRNA-150 in the blood samples, we carried out the serum samples detection experiment. Five different concentrations of artificial synthesized miRNA-150 were spiked in tenfold diluted human health serum and tested with the strategy we designed. Recovery rates were listed in Table 1 ranged from 92.7% to 106.9%, implying that the miRNA-150 in spiked serum samples could be detected by this method.

**Table 1:** Actual and measured concentration of miRNA-150 in human serum samples

Sample number	Added miRNA	Measured miRNA	RSD (%) (n = 6)	Recovery (%)
1	100 pM	104.36 pM	6.19	104.36
2	10 pM	10.69 pM	2.86	106.9
3	1 pM	0.97 pM	6.10	97
4	100 fM	102.11 fM	4.00	102.11
5	10 fM	9.27 fM	3.90	92.7

Moreover, the efficiency of our sensor has also been proved by serum samples from NSCLC patients and healthy people, and qRT-PCR confirmed this result. The Ethics and Clinical Research Committee of the Southwest Hospital of Third Military Medical University (Army Medical University) Group approved our research, and all contributors have signed written informed consents. From healthy contributors and NSCLC patients, we obtained the serum samples. Levels of miRNA-150 in the serum samples were direct quantified with the proposed assay and qRT-PCR respectively. Figure 4c showed the real human samples results for both NSCLC patients and healthy contributors, the similar tendency as the results of qRT-PCR. The results of probe method were obtained within 1 h, but qRT-PCR assays required more than 3 h procedures.

The proposal method successfully used for the quantification of miRNA-150 on laboratory and real samples from healthy contributors and NSCLC patients. These results suggested that the established miRNA probe holds potential application in miRNA detection of real samples.

### Specificity of the allosteric spherical nanoprobe

**Fig. 5:**
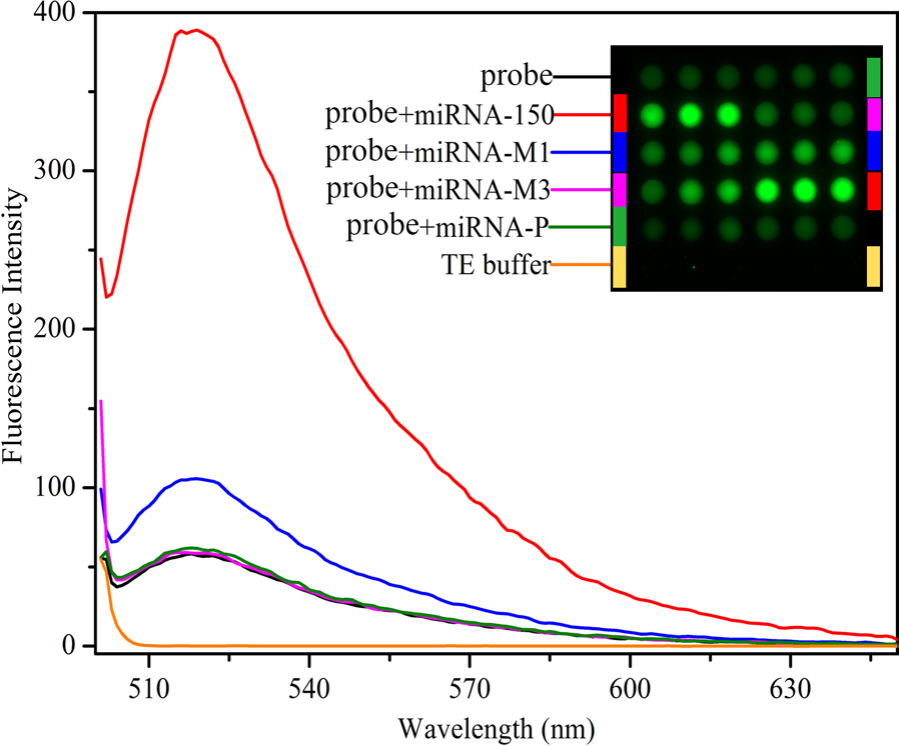
Selectivity of the proposed method. Fluorescence emission spectra in response to miRNA-150, single-base mismatched miRNA-150 (miRNA-M_1_), three-base mismatched miRNA-150 (miRNA-M_3_) and a completely unrelated sequence (miRNA-P). Inset shows the multipoint fluorescence scanning results on microarray corresponding to the fluorescence emission spectra

The increased burden of diagnosing for NSCLC is largely due to the high sequence homology of miRNA. Therefore, it should be emphasized that the specificity presented in this method is available. Figure 5 shows the comparison of fluorescence spectra in response to different kinds of miRNAs targets under the optimal conditions. Notably, the fluorescence intensity in response to miRNA-150 is approximately 3.7-fold higher than that in response to single-base mismatched miRNA-150 (miRNA-M_1_) and approximately 6.64-fold higher than that in response to three-base mismatched miRNA-150 (miRNA-M_3_) and a completely unrelated sequence (miRNA-P). Inset shows the multi-point fluorescence scanning results on microarray. These results demonstrate that the proposed method has high selectivity in discriminating miRNA-150 from analogous miRNA due to the structure of the loop-stem.

## Conclusions

In brief, a rapidly, specifically and sensitively allosteric spherical nanoprobe for the assay of miRNA-150 was developed, without the involvement of any other complicated amplification technology. The sensing design depends on the allosteric spherical nanoprobe, which enable to convert the specific miRNA-150 recognition reactions into measurable fluorescence signals. It exhibits high sensitivity toward NSCLC-related miRNA-150 with a detection wide range of 6 orders of magnitude and a detection limit as low as 38 fM. The double loop-stem structure of the nanoprobe could identify single-base mismatched target. More importantly, the proposed method can directly distinguish the expression of serum miRNA-150 NSCLC patients and healthy people. Although much remains to be studied about the performance of the sensing in complex biological, this probe paves the way for further applications in the clinical diagnosis of lung cancers. We hope that this allosteric spherical nanoprobe can be used for bioanalysis, especially in the field of POCT.

## Supplementary information

**Additional file 1: Fig S1**. Schematic diagram of microarray structure.** Table S1**. Alignment of sequences used in experiment.** Table S2.** Comparison of the reported chemosensors for miRNA.

## Data Availability

All data generated or analyzed during this study are included in this published article.
