# Supplementary material for: A novel quantification platform for point-of-care testing of circulating MicroRNAs based on allosteric spherical nanoprobe
Source: J Nanobiotechnology. 2020 Oct 31;18:158. doi: 10.1186/s12951-020-00717-z (PMC7603675; doi:10.1186/s12951-020-00717-z)
Supplement: Supplementary file 1 — Additional file 1: Fig S1. Schematic diagram of microarray structure. Table S1. Alignment of sequences used in experiment. Table S2. Comparison of the reported chemosensors for miRNA. [file 12951_2020_717_MOESM1_ESM.docx]

**Additional file 1**

**A Novel Quantification Platform for Point-of-care Testing of Circulating MicroRNAs Based on Allosteric Spherical Nanoprobe**

Huiyan Tian^a#^, Changjing Yuan^a#^, Yu Liu^a^, Zhi Li^a^, Wei Liu^a^, Ke Xia^a^, Mengya Li^a^, Fengxin Xie^a^, Qinghai Chen^a^, Ming Chen^a^, Weiling Fu^a^* & Yang Zhang^b^*

^a^ Department of Laboratory Medicine, First Affiliated Hospital, Third Military Medical University (Army Medical University), Chongqing, China

^b^ Department of Laboratory Medicine, Chongqing University Cancer Hospital, Chongqing, China

# The authors contributed equally to this work.

* Corresponding author:

Weiling Fu, MD, PhD. E-mail: fwl@tmmu.edu.cn, Tel: (86)13708309571.

Yang Zhang, MD, PhD. E-mail: [millen001@163.com](mailto:millen001@163.com), Tel: (86)18512371269.


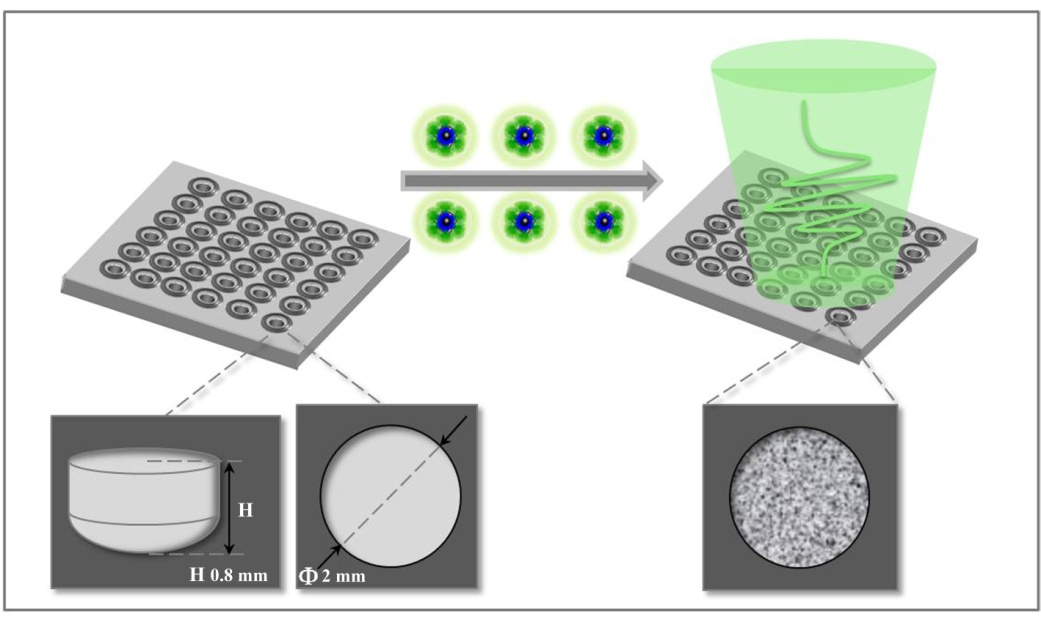


**Fig S1** Schematic diagram of microarray structure

**Table S1. Alignment of sequences used in experiment**

| Sequence name | Sequence from 5′ to 3′ | Length (nt) |
| --- | --- | --- |
| Seq A | FAM-CGCGATTCTCCCAACCCTTGTACCAGTGATCGCGTGAGCA  CATGAAATACACTGGAGAATCG-BHQ1 | 62 |
| Seq B | FAM-ATGCGCTCTCCCAACCCTTGTACCAGTGGCGCATCGATTC  TCCAGATCCCACAGTGATGTAC-NH_2_ | 62 |
| Seq C | CACTGTGGGATTGTATTTCATGTGCTCA-BHQ1 | 28 |
| miRNA-150 | CACUGGUACAAGGGUUGGGAGA | 22 |
| miRNA-M_1_  miRNA-M_3_ | CACUGGUACAAAGGUUGGGAGA  CACGGGUACAAAGGUUAGGAGA | 22  22 |
| miRNA-P | ACAGUUGCACCUUUGGUUUCUC | 22 |

**Table S2. Comparison of the reported chemosensors for miRNA**

| Detection method | Strategy ^a^ | Dynamic range | Limit of detection | Detection time | References |
| --- | --- | --- | --- | --- | --- |
| Electrochemical detection | RCA-mediated PbNPs | 50 aM to 100 fM | 8.6 aM | More than 4 hours | [[1](#_ENREF_1)] |
| Colorimetric detection | Metal chelator labeled oligonucleotide probe | 10-100 fM | 8.9 fM | More than 3 hours | [[2](#_ENREF_2)] |
| Surface plasmon  resonance | Enzyme-assisted target recycling system | 5 pM to 10 nM | 2.45 pM | More than 3 hours | [[3](#_ENREF_3)] |
| Surface-enhanced Raman scattering | CHA | 10 fM to 100 nM | 3.5 fM | Within 1 h | [[4](#_ENREF_4)] |
| Fluorescence detection | HP-RCA | 0.2 fM to 1 nM | 10 fM | More than 5 hours | [[5](#_ENREF_5)] |
| Fluorescence detection | DSN-RCA | 5 pM to 5 nM | 1.5 pM | More than 3 hours | [[6](#_ENREF_6)] |
| Fluorescence detection | double-hairpin spherical nanoprobe | 100 fM to 10 nM | 38 fM | Within 1 h | This method |

^a^ RCA-mediated PbNPs: rolling circle amplification (RCA)-mediated palladium nanoparticles

CHA: catalytic hairpin assembly

HP-RCA: hairpin probe-based rolling circle amplification

DSN-RCA: duplex-specific nuclease mediated target recycling amplification

References

[1] Cuiling Zhang, Dan Li, Dongwei Li, Kai Wen, Xingdong Yanga, Zhu, Y.(2019)Rolling circle amplification-mediated in situ synthesis of palladium nanoparticles for the ultrasensitive electrochemical detection of microRNA. The Analyst 144:3817-3825. doi:10.1039/c9an00427k.

[2] Jiafang Piao, Qian Zhao, Dianming Zhou, Weipan Peng, Weichen Gao, Minghui Chen, Guiming Shu, Xiaoqun Gong, Chang, J.(2019)Enzyme-free colorimetric detection of MicroRNA-21 using metal chelator as label for signal generation and amplification. Analytica Chimica Acta 1052:145-152. doi:10.1016/j.aca.2018.11.04.

[3] Jisun Ki, Hyo young Lee, Hye Young Son, Yong-Min Huh, Haam, S.(2019)Sensitive Plasmonic Detection of miR-10b in Biological Samples Using Enzyme-Assisted Target Recycling and Developed LSPR Probe. ACS Applied Materials & Interfaces 11:18923-18929. doi:10.1021/acsami.9b03005.

[4] Wenbin Cheng, Ye Zhang, Hua Yu, Wei Diao, Fei Mo, Bo Wen, Wei Cheng, Yan, Y.(2018)An enzyme-free colorimetric biosensing strategy for ultrasensitive and specific detection of microRNA based on mismatched stacking circuits. Sensors and Actuators B: Chemical 255:3298-3304. doi:10.1016/j.snb.2017.09.157.

[5] Y. Li, Liang, L., Zhang, C.Y.(2013)Isothermally sensitive detection of serum circulating miRNAs for lung cancer diagnosis. Analytical chemistry 85:11174-11179. doi:10.1021/ac403462f.

[6] Lin Tan, Liu Xu, Jin-Wen Liu, Li-Juan Tang, Hao Tang, Yu, R.(2019)Duplex-specific nuclease-mediated target recycling amplification for fluorescence detection of microRNA. Analytical Methods 11:200-204. doi:10.1039/c8ay02265h.
